# Hantavirus Induced Kidney Disease

**DOI:** 10.3389/fmed.2021.795340

**Published:** 2022-01-18

**Authors:** Sheema Mir

**Affiliations:** College of Veterinary Medicine, Western University of Health Sciences, Pomona, CA, United States

**Keywords:** hantavirus, HFRS, acute kidney injury, bunyavirus, hemorrhagic fever

## Abstract

Hantavirus induced hemorrhagic fever with renal syndrome (HFRS) is an emerging viral zoonosis affecting up to 200,000 humans annually worldwide. This review article is focused on recent advances in the mechanism, epidemiology, diagnosis, and treatment of hantavirus induced HFRS. The importance of interactions between viral and host factors in the design of therapeutic strategies is discussed. Hantavirus induced HFRS is characterized by thrombocytopenia and proteinuria of varying severities. The mechanism of kidney injury appears immunopathological with characteristic deterioration of endothelial cell function and compromised barrier functions of the vasculature. Although multidisciplinary research efforts have provided insights about the loss of cellular contact in the endothelium leading to increased permeability, the details of the molecular mechanisms remain poorly understood. The epidemiology of hantavirus induced renal failure is associated with viral species and the geographical location of the natural host of the virus. The development of vaccine and antiviral therapeutics is necessary to avoid potentially severe outbreaks of this zoonotic illness in the future. The recent groundbreaking approach to the SARS-CoV-2 mRNA vaccine has revolutionized the general field of vaccinology and has provided new directions for the use of this promising platform for widespread vaccine development, including the development of hantavirus mRNA vaccine. The combinational therapies specifically targeted to inhibit hantavirus replication and vascular permeability in infected patients will likely improve the disease outcome.

## Introduction

Viral hemorrhagic fever refers to a multisystem syndrome triggered by severe damage to the vascular system by the viruses from six distinct families: *Filoviridae, Arenaviridae, Hantaviridae, Nairoviridae, Phenuiviridae*, and *Flaviviridae* (Table 1). The disease symptoms are accompanied by fever and hemorrhage (bleeding), although the bleeding by itself is hardly ever life-threatening. These enveloped RNA viruses are carried by animal or arthropod vectors in nature. Humans are infected by contact with infected hosts or their contaminated body fluids such as saliva, feces, or blood. The mode of transmission and severity of the disease depends upon virus species, although each can cause hemorrhagic fever. Outbreaks of viral hemorrhagic fever are sporadic and their occurrences are not easily predictable. Based on certain characteristics such as morbidity and mortality, the possibility of person-to-person transmission, aerosolic dissemination, availability of vaccine or therapeutic treatments, stability in the environment, and potential for large scale production etc, some of the hemorrhagic fever viruses have been classified as potential bio-warfare agents. These viruses include Ebola, Marburg, Lassa fever, Machupo, Junin, Guanarito, Sabia, Rift valley fever, yellow fever, Omsk hemorrhagic fever, and Kyasanur forest disease (1). Among other hemorrhagic fever viruses (Table 1), the old-world hantaviruses are known to cause hemorrhagic fever with renal syndrome (HFRS), a group of clinically similar illnesses targeting the kidney.

**Table 1:** Hemorrhagic fever virus.

**Family**	**Virus**	**Disease**
*Filoviridae*	Ebola^1^, Marburg	Ebola HF, Marburg HF
*Arenaviridae*	Lassa, NW Arenaviruses^2^	Lassa fever, NW2 hemorrhagic fever.
*Nairoviridae*	CCHFV^3^	CCHF hemorrhagic fever
*Phenuiviridae*	RVFV^4^	Rift valley fever
*Hantaviridae*	New word hantavirus	Hantavirus cardiopulmonary syndrome
	**Old world hantavirus**	**Hemorrhagic fever with renal syndrome**
*Flaviviridae*	Dengue	Dengue fever, Dengue HF, Dengue SY^5^
	YFV^6^	Yellow fever
	Omsk HFV^7^	Omsk hemorrhagic fever
	Kyasanur FDV^8^	Kyasanur forest disease

1 *There are four subtypes of Ebola (Zaire, Sudan, Ivory Coast and Reston), Ebola HF stands for Ebola hemorrhagic fever and Marburg HF stands for Marburg hemorrhagic fever*.

2 *Stands for New word ArenavirusesThe new word Arena viruses include (Machupo, Junin, Guanarito, Sabia)*.

3 *Crimean Congo Hemorrhagic Fever Virus*.

4 *Rift Valley Fever Virus*.

5 *Dengue shock syndrome*.

6 *Yellow Fever Virus*.

7 *Osmak Hemorrhagic Fever Virus*.

8 *Kyasanur Forest Disease Virus. The information presented in this table was obtained from (1)*.

Hantaviruses are emerging negative strand RNA viruses and members of the *Hantaviridae* family (2–4). They are carried by rodents, and humans get infected by the inhalation of aerosolized excreta such as saliva and urine droppings of infected rodent hosts (5–8). Their infection causes a significant impact on human health (8, 9). Hantavirus species such as Puumala virus (PUUV), Seoul virus (SEOV), Dobrava Belgrade virus (DOBV), and hantaan virus (HTNV) are predominantly found in Asia and Europe and are referred to as old world hantaviruses (Table 2). The hantavirus species such as Sin Nombre virus (SNV) and Andes virus (ANDV) are mostly found in America are referred to as new world hantaviruses. Old and new world hantaviruses have distinct pathologies. Old world hantaviruses infect the highly specialized and differentiated endothelial cells of the kidney, causing acute renal failure with tubular and glomerular involvement, which is referred to as hemorrhagic fever with renal syndrome (HFRS). The new world hantaviruses cause hantavirus cardiopulmonary syndrome (HCPS) (24), a fibril illness characterized by respiratory failure and cardiac dysfunction. The mortality rates of HFRS and HCPS can go as high as 15 and 50%, respectively, in certain outbreaks (25, 26). Annually, 150,000 to 200,000 cases of hantavirus infection are reported worldwide (27), and more than 50,000 reported cases are found in China alone. There is no FDA approved vaccine or an antiviral therapeutic against hantavirus infections.

**Table 2:** Old world hantaviruses species.

**Virus**	**Host**	**Geographical distribution/reference**
Hantaan (HTNV)	Striped field mouse	Asia (10)
Seoul (SEOV)	Rat worldwide	Worldwide (11)
Dobrava (DOBV)	Yellow-necked mouse	Europe (9)
Saaremaa (SAAV)	Striped field mouse	Europe (12)
Thailand (THAIV)	Bandicoot rat	Thailand (13)
Amur (AMRV)	Korean field mouse	Asia (14)
Puumala (PUUV)	Bank vole	Europe (15)
Hokkaido (HOKV)	Red bank vole	Asia (16)
Tula (TULV)	European common vole	Europe/Russia (17)
Prospect Hill (PHV)	Meadow vole	North America (18)
Bloodland Lake (BLLV)	Prairie vole	North America (19)
Isla Vista (ISLAV)	Californian vole	North America (20)
Khabarovsk (KHAV)	Reed vole	Asia/East Russia (21)
Topografov (TOPV)	Lemming	Siberia/Russia (22)
Thottapalayam (TPMV)	Shrew	Asia/India (23)

**Figure 1:**
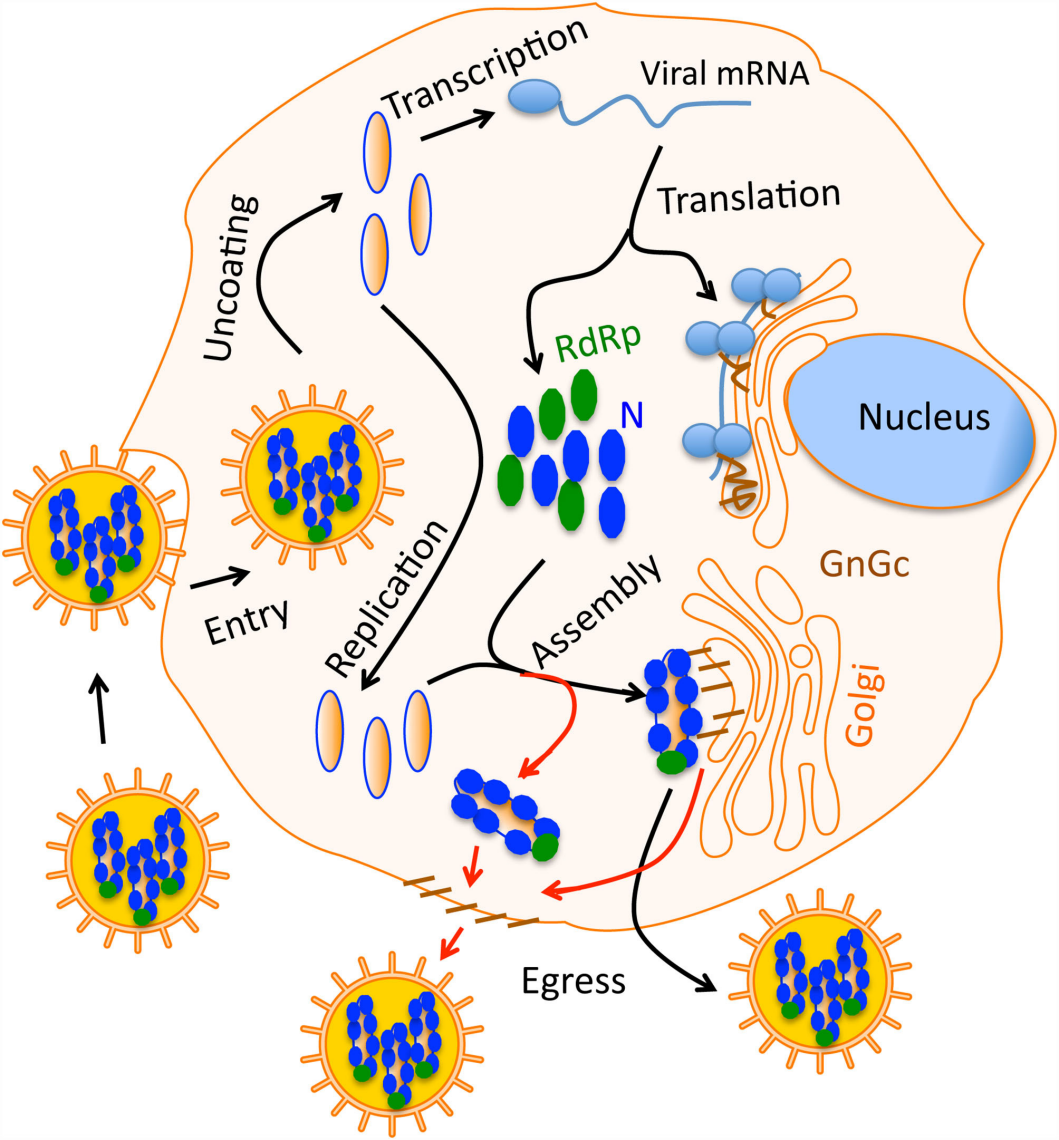
A simple graphical sketch of the hantavirus replication cycle. Hantavirus particles harboring the three nucleocapsids (blue) bind the host cell surface receptor. After entry virus uncoating takes place and capped viral mRNAs are synthesized by transcription. Viral RdRp replicates the viral genome. Viral proteins are synthesized by the host translation machinery. Glycoprotein Gn and Gc are transported to Golgi. During virus assembly, the nucleocapsids meet the glycoprotein on the Golgi surface and new virus particles are born inside the Golgi, which then egress the cell through secretary mechanisms. In some hantaviruses, the assembly occurs on the host cell membrane (red line). In this case, nucleocapsids meet the glycoprotein on the cell membrane that are transported through Golgi.

Under an electron microscope hantavirus particles appear spherical in shape (28). The three hantaviral genomic RNA segments: S, M, and L encode viral nucleocapsid protein (N-protein), glycoprotein precursor (GPC), and viral RNA dependent RNA polymerase (RdRp), respectively. The GPC precursor is post-translationally cleaved in the middle generating two glycoprotein Gn and Gc that are incorporated in the virus envelop (29). Hantaviruses primarily target endothelial cells with the receptor (β3 integrin) for virus attachment and entry. Hantaviruses use surface glycoproteins to attach to the cell surface receptor of the target cell (Figure 1). The endothelial cells make the internal linings of blood vessel walls, making the body's vascular system susceptible to viral infection. Hantavirus replication occurs exclusively in the host cell cytoplasm (Figure 1). Immediately after entering into the host cell, viral uncoating occurs and viral RdRp initiates transcription by a unique cap snatching mechanism to generate 5' capped viral mRNAs (30–32). The multifunctional N-protein plays diverse roles in the virus replication cycle. It is involved in viral transcription initiation in conjunction with viral RdRp, facilitates mRNA translation, and encapsidates the viral genome (33–36). Since this article is mainly focused on hantavirus induced HFRS that leads to AKI, a brief overview of AKI is presented below, followed by discussion of hantavirus induced HFRS leading to AKI. Consistent with the objectives of this journal, the review article provides a link between basic research and clinical practice, with special emphasis on studies that are directly relevant to patient care.

### Acute Kidney Injury (AKI)

AKI refers to the rapid decrease in the renal filtration function of the kidney. The condition is primarily observed by increased levels of blood urea nitrogen (BUN) and creatinine. AKI is a general healthcare problem affecting up to 40% of patients admitted to critical care hospital units (37). Apart from predisposition risk factors, the degenerative processes affecting renal epithelium and vasculature play an important role in AKI (38). Moreover, innate and adaptive immune responses impacting renal epithelium and vasculature functions also contribute to AKI (38). Apoptosis and necrosis of tabular epithelium can lead to nephron loss accompanied by the activation of the immune response, resulting in the decline of the kidney's filtration capacity (39). The increased chemokine and cytokine expression along with elevated innate and adaptive immune cell response are observed during renal ischemia, another major cause of AKI (39). However, the T-regulatory cell (Treg) response prevents kidney tissue damage by suppressing the inflammatory response to self-antigens (40). Oxidative stress is another leading cause of AKI. Mitochondrial dysfunction due to renal ischemia may lead to increased production of reactive oxygen species (ROS), promoting AKI due to acute tubular necrosis (40). The use of mitochondria specific ROS scavenger (Mito-TEMPO) (41) and stimulation of mitochondria biogenesis by formoterol has been reported to improve AKI in animal models (42). Thus, selective improvement in mitochondrial function can reduce kidney injury and ultimately reverse AKI. Endoplasmic reticulum (ER) stress, occurring due to the accumulation of misfolded proteins in the ER can also lead to AKI. The stress can be relieved by the expression of molecular chaperons such as, heat shock proteins that transiently bind the misfolded target proteins and help them to refold correctly and attain proper biological function. The unrelieved ER stress has been shown to generate reactive oxygen species (ROS) that ultimately lead to AKI by apoptosis or acute tubular necrosis (43). Nephrotoxic drugs such as tuncamycin have been reported to induce ER stress due to protein misfolding (44). Induction of heat shock protein expression in AKI rodent models has been reported to improve AKI by preventing tubular necrosis (45). The induced expression of pro-apoptotic mediators CHOP/GADD153 due to severe ER stress is consistent with the observed loss of nephron epithelial cells by apoptosis during AKI (46, 47). The use of a chemical chaperone 4-phenylbutyrate reduced both the CHOP/GADD153 protein expression and tubular necrosis in nephrotoxin induced AKI mouse models (44). ER stress inhibitors such as 4-phenylbutyrate have demonstrated efficacy in reducing AKI in preclinical trials (44, 48). The endothelium regulates the blood flow to the local tissue beds and modulates numerous processes related to coagulation, inflammation and vascular permeability (38). The severe impact on endothelium due to AKI leads to microvasculature dysfunction, causing further injury and complications in renal function (49, 50). Due to limited regenerative power of per-tubular capillaries, the endothelial damage due to AKI leads to their rarefaction, causing interstitial fibrosis and increased risk of chronic kidney disease (CKD) (39, 49–51).

## Hantavirus Induced HFRS Leads to AKI

The old-world hantaviruses primarily target the kidney, explaining why the hantavirus disease was initially called “nephropathia epedemica (NE)” in the western world. The kidney tropism and molecular mechanism of NE remain poorly understood. Later WHO started to refer to the old world hantavirus disease as HFRS, although the term HFRS is most popular in Asia and eastern Russia where the disease due to Hantaan virus (HTNV) species is more severe compared to Puumala virus (PUUV) induced NE in Europe and western Russia (52). Hantavirus induced HFRS is listed as one of the fifteen major factors leading to acute kidney injury (AKI) in the Western world (53, 54). Both HCPS and HFRS patients present non-specific flue-like symptoms such as fever, headache, abdominal pain, malaise, and nausea to the clinic. This early febrile phase may last for 2–3 days and is followed by a hypotensive phase in which patients present severe thrombocytopenia, elevated levels of lactate dehydrogenase, C-reactive protein, increased vascular leakage, and leukocytosis (5). Thrombocytopenia was observed in 80% of documented PUUV infections and is even more frequent in other HFRS causing viruses such as HTNV, DOBV, and SEOV. In the 1996 NE outbreak in Belgium, the platelet level at the time of patient's admission to the clinic was reported below 150,000/ml in 79% of 217 infected patients (55). After the hypotensive phase, the oliguric phase begins during which viral infection manifests in different organs. In HCP patients, cardiopulmonary involvement is predominantly observed although renal symptoms cannot be completely ruled out. However, HFRS and NE selectively impact the kidney. The laboratory examination of HFRS and NE patient samples shows proteinuria and high serum creatinine concentrations. The urinalysis shows hematuria and albuminuria (5). Proteinuria is a constant sign in all HFRS and HCPS patients, even though HCPS does not primarily target the kidney (56). Proteinuria has been reported in 100% of HCPS cases. The proteinuria in HFRS can be as high as 29 g/L (56), and some severely ill patients may require dialysis. The severe kidney injury by DOBV infection prompted dialysis in 30% of infected patients in an outbreak in Greece (57). Due to their high frequency, a case presentation without early thrombocytopenia and proteinuria is likely not a hantavirus case, even in HCP patients (56). Acute renal failure (ARF) in the oliguric phase is observed in 90–95% of HFRS patients infected with old world hantaviruses, although the ARF due to PUUV induced NE can be mild (5). An examination of 217 patients in PUUV induced NE outbreak in 1996 in Belgium revealed that 70% of infected patients developed ARF with serum creatinine levels ranging between 1.6 to 20.72 mg/dl (55). Acute myopia is another most common presenting sign in about 25% of NE cases (58). This early transient ophthalmic sign is very specific for old world hantavirus infections due to its absence in other acute infections mimicking HFRS (56). The oliguric phase is followed by the diuretic phase in which high proteinuria rapidly starts to decrease and renal function gradually improves. The proteinuria lasting for years due to hantavirus infection has never been previously demonstrated convincingly (59). However, a recent follow-up study (7–35 months) on 456 PUUV infected patients in Germany revealed hematuria, hypertension, and proteinuria in 25, 23, and 7% patients, respectively (60). NE-associated hypertension and proteinuria do not appear to be concerning in the long run, but NE-associated hematuria might (60). During convalescent phase patients completely recover. Due to a favorable prognosis, the mortality rate of PUUV induced NE is <1% (61), although long term hypertension and hematuria due to PUUV infection are being discussed (62). The mortality rate of 5–15% in HFRS is likely due to several complications including renal insufficiency, edema, hemorrhages, encephalopathy, and shock (5). Although the predisposition factors may impact the hantavirus disease outcome, the severity of illness mostly depends upon the hantavirus species causing the infection (11).

### Mechanism of Hantavirus Induced AKI

**Figure 2:**
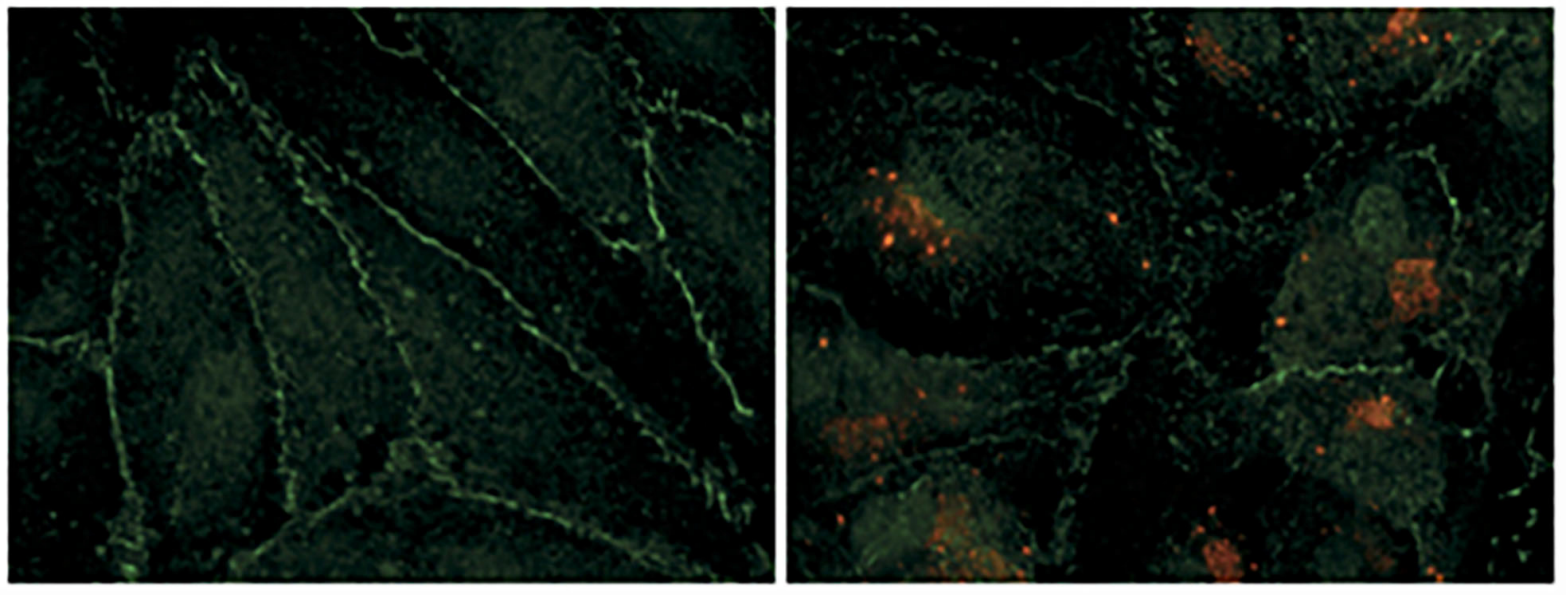
Hantavirus infection damages the contacts between endothelial cells. Human renal glomerular endothelial cells were infected with puumala hantavirus. Cells were examined by immunofluorescence microscopy. Hantavirus nucleocapsid protein is shown by red color and the tight junction marker protein (ZO-1) is shown by green color. The uninfected cells on the left show well-organized cell-to-cell contacts evident from continuous peripheral staining of ZO-1. The uninfected cells form an intact monolayer. The virus-infected cells on the right display discontinue ZO-1 staining, demonstrating the breakdown of endothelial barrier function. This picture was borrowed from (5) and is reused with permission from the Nature publishing group.

**Figure 3:**
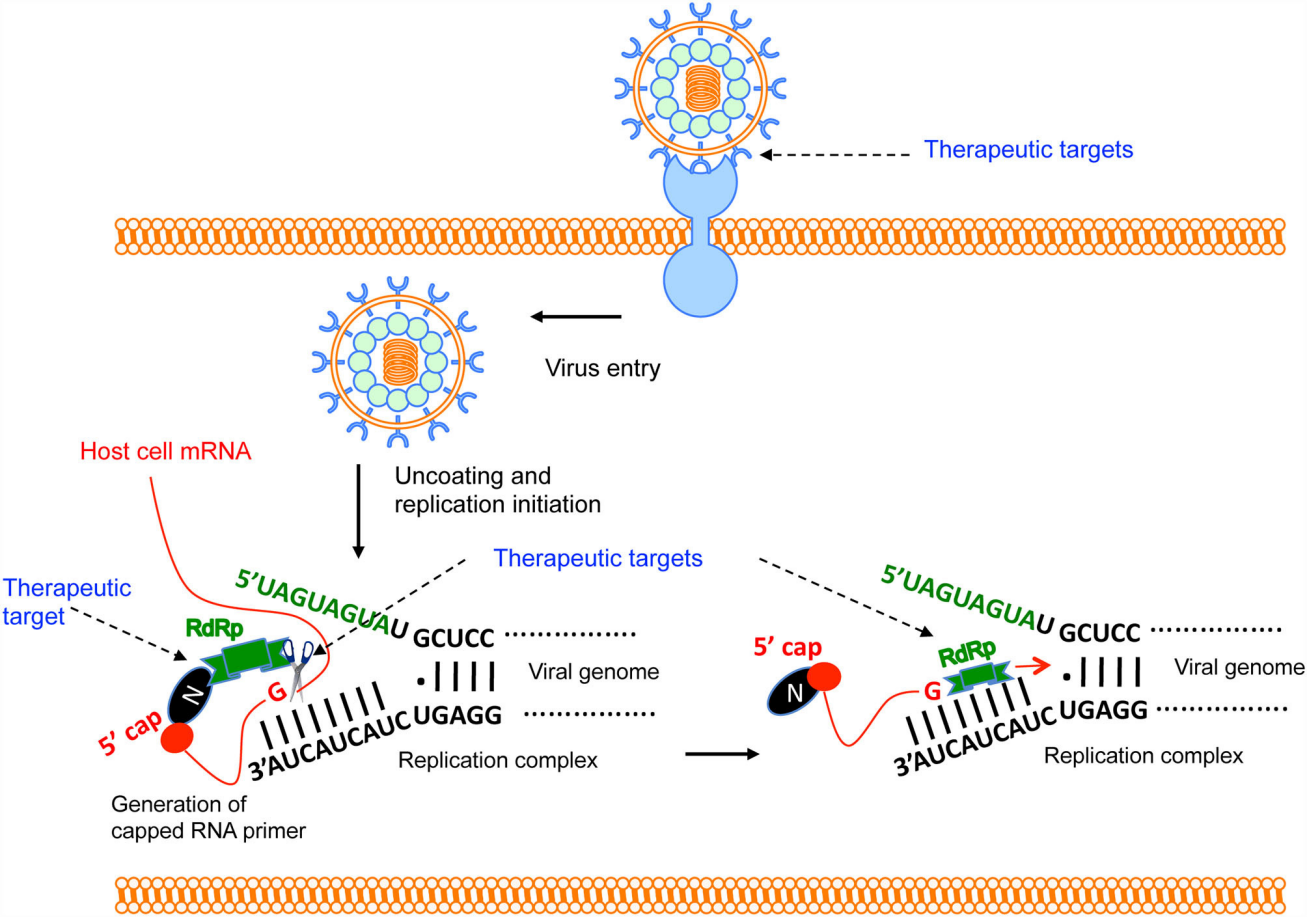
Brief overview of Hantavirus replication cycle and therapeutic targets. The virus binds to the host cell's receptor. After entry, virus uncoating takes place and virus replication is initiated. N protein binds to the host mRNA caps (36). RdRp binds to the N protein through C-terminus (33). The N-terminal endonuclease domain of RdRp cleaves the host cell mRNA at a “G” residue 14 nucleotides downstream of the 5′ cap to generate the capped RNA primer (30). The primer anneals with the 3′ terminus of the viral genome and transcription is initiated by the prime and re-align mechanism. Potential therapeutic targets are shown by the arrow.

**Figure 4:**
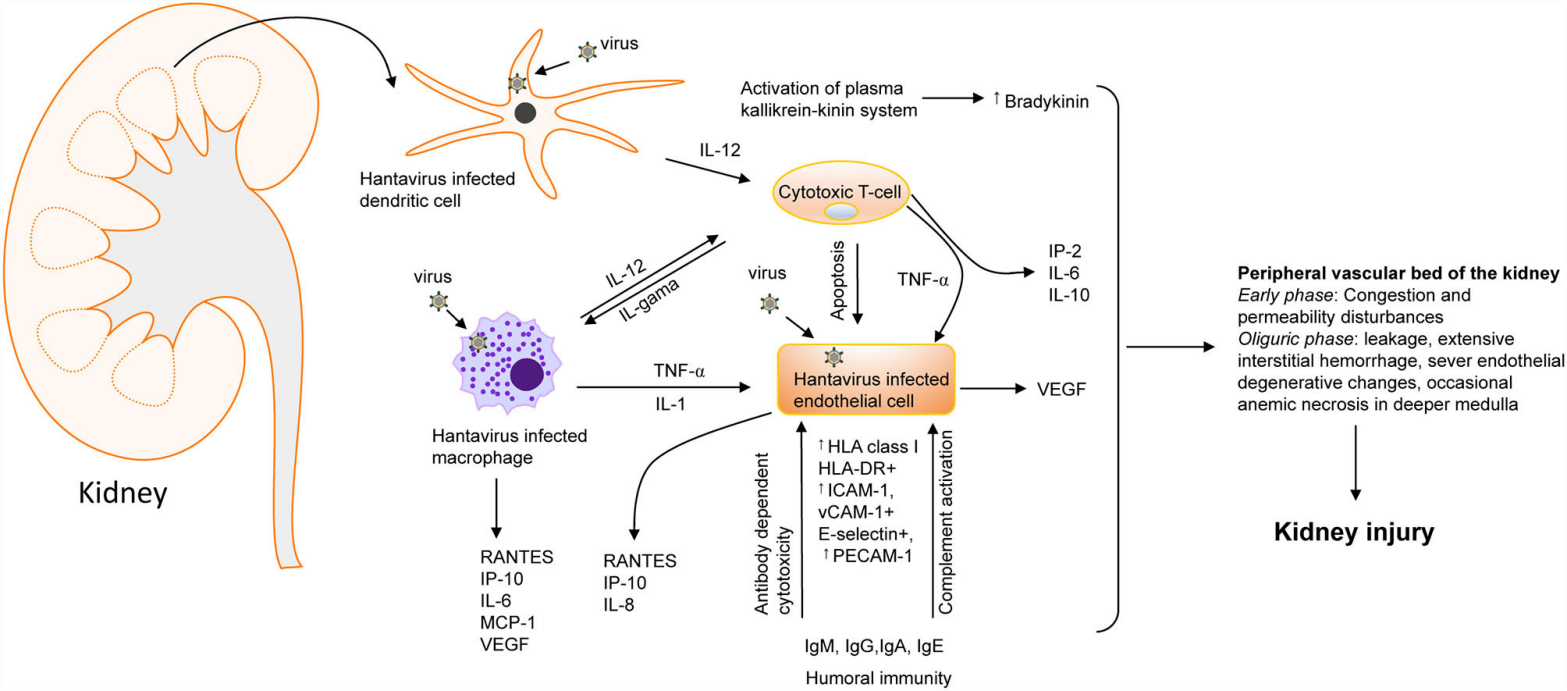
Hantavirus induced kidney injury. A flow chart showing the involvement of cytokines [IL-1, IL-2, IL-6, IL-10, IL-12, TNF-, INF-, and vascular endothelial growth factor (VEGF) and chemokines] [RANTES, monocyte chemoattractant protein-1 (MCP-1), IP-10, and IL-8. ICAM-1, intercellular adhesion molecule-1; PECAM-1, platelet-endothelial cell adhesion molecule-1; VCAM, vascular cell adhesion molecule-1] in hantavirus induced kidney injury (64). Increased bradykinin levels can also trigger cytokine storms during hantavirus infection (65). the most severe vascular affection includes congestion and permeability disturbances during the early phases, followed by severe blood stasis accompanied by leakage, extensive interstitial hemorrhage, severe endothelial degenerative changes, and occasionally anemic necrosis in the deeper medulla that culminate into kidney injury (64, 66–69).

The clinical description of HFRS is an acute renal failure with significantly elevated proteinuria caused by tubular and glomerular involvement. The interdisciplinary research approaches from molecular virology, epidemiology, and nephrology have provided crucial insights into the pathogenesis of hantavirus infection. The mechanism of kidney injury appears immunopathological, characterized by deterioration of endothelial cell function and compromised barrier functions of the vasculature, likely due to cytokine storm in infected patients during the virus infection (Figure 2). Infection of human renal cells critical for barriers functions of the kidney such as podocytes, tubular epithelial and glomerular endothelial cells revealed disturbances in structure and integrity of cell to cell contacts, observed by redistribution and reduction of the light junction protein ZO-1 along with decreased transepithelial resistance in infected epithelial monolayers (63) (Figure 4). The in-depth molecular details of hantavirus induced AKI remain poorly understood. As the human leukocyte antigen (HLA) haplotypes were found to play a role in the outcome of hantavirus disease (70–72), the severity of hantavirus infection in certain endemic areas may likely be influenced by the genetic susceptibility due to the prevalence of certain HLA genes in the inhabitant population (5). The relationship between HLA alleles and disease severity suggests the involvement of T-cell mediated immune response in hantavirus infection. This is supported by the observations of elevated CD8+ cell count in HCPS and HFRS patients (73, 74). The characteristic feature of hantavirus induced AKI is the increased vascular permeability without apoptotic damage to the capillary endothelium, suggesting the likely breakdown of endothelium due to cytokine release (Figure 3). This immunological rather than anatomical insult to the endothelium is reflected by the scarcity of renal lesions on kidney biopsies (56). The observed lesions are largely normal except interstitial edema sometimes accompanied with patchy monocellular infiltrate can be noted. The lesions with interstitial microhemorrhages are very rare and exceptional (52, 56). The primary function of the endothelium is to regulate vascular permeability. However, upon hantavirus infection, the endothelial cells up-regulate certain signaling pathways and induce the expression of proinflammatory cytokines, thereby manifesting the amplified immune response for the rapid recruitment of immune cells at the site of inflammation (Figure 4). The vigorous immune response activates the compliment system and triggers the release of proinflammatory cytokines that interfere in endothelial cell function and likely induce vascular permeability. Although numerous cytokines are released in humans (75), the identification of cytokines mediating the vascular leakage could provide new directions for therapeutic strategies of hantavirus induced AKI. T-regulatory cells (TRegs) on the other hand are known to prevent kidney injury by suppressing the proinflammatory response. Interestingly, the TReg response is down-regulated in humans during hantavirus infection (76–78), which may likely contribute to the inflammation-mediated AKI in hantavirus infected patients. In contrast, the up-regulated Treg response promotes hantavirus persistence in infected rodent hosts (79). Although the elevated levels of T cells and cytokine producing cells in hantavirus infected patients support the cytokine induced vascular leakage during hantavirus AKI (80), a recent study demonstrated that depletion of T cells did not impact the outcome of hantavirus disease in a Syrian hamster model (81).

Another hypothesis of increased vascular leakage during hantavirus induced AKI stems from the observations that over-expressed vascular endothelial growth factor (VEGF) could impact vascular permeability by promoting the degradation of VE-cadherin (81–84). VE-cadherin is an important adhesion molecule facilitating intracellular contacts between endothelial cells and regulating vascular permeability (85). One more *in vitro* study has suggested the role of the kallikrein-kinin system (KKS) in the vascular leakage of hantavirus infected patients (65). Activation of this system results in the release of a nine amino acid long peptide called bradykinin (BK) (86). The BK is an extremely potent inflammatory molecule that plays an active role in the vasodilation and increased permeability of the vasculature (87–89). The *in vitro* finding of increased KKS activation, clinical studies demonstrating activation of prekallikrein (an intermediate in the KKS cascade), and successful treatment of PUUV infected HFRS patients using BK antagonists suggest that KKS activation and release of BK might play a role in the hantavirus induced AKI (65, 90).

### Epidemiology of Hantavirus Induced Kidney Injury

The epidemiology of hantavirus induced kidney injury is related to the hantavirus species and the geographical distribution of the natural host carrying the virus (Table 2). Mostly, the hantavirus induced AKI is caused by old world hantaviruses born out of *Myodes, Rattus*, and *Apodemus* rodents. Hantavirus infections in other animals such as shrews, bats, and moles are considered spillover infections and there is little information about their transmission and severity of disease in humans (Table 2) (91). The chances of acquiring the disease are based on the exposure of humans to rodents or their infected excreta in the endemic zones of the disease. The human-human transmission has not been reported in old world hantaviruses. Moreover, the human - rodent contacts are influenced by numerous factors such as climate changes and disturbances in rodent habitats by deforestation may favor the migration of rodents to human dwellings [discussed in detail in (27)].

Hantan virus (HTNV) and Seoul virus (SEOV) infections are mostly found in Eurasia, especially in China, south Korea, east Russia, and northern Europe. China has the highest HFRS case load in the world, accounting for more than 90% of the total number of HFRS cases worldwide (92). From 2006 to 2012 a total number of 77,558 HFRS cases and 866 deaths were reported in China alone. More than 90% of these cases were clustered in nine provinces and mainly reported in the spring and autumn seasons (93). Observed SEOV infection in urbanized cities has put an end to the earlier thoughts that hantavirus infection is a rural disease (63, 94–96). PUUV associated AKI is found throughout the European content within the range of *Myedous Glareolus* habitat. In Europe, 35,424 cases of PUUV were reported by the end of 2006, although most of these cases were reported to have an origin from Finland (63, 97, 98). Other countries having significant cases of PUUV kidney injury include Sweden, Belgium, France, Germany, and Norway (97). The DOBV infections are most common in the Balkan region, although both PUUV and DOBV seroprevalence is reported in different Balkan countries including Bosnia, Greece, Slovenia (99, 100). Hantavirus induced HFRS likely occur in other Asian countries as the hantavirus antibodies have been found in rodents and humans in Thailand (101, 102), Indonesia (103, 104), and India (105). The epidemiological studies have revealed that males are more prone to hantavirus infection as compared to females. The male: female disease ratios vary from 2–5:1. However, the fatality rates of infected females are higher compared to males (106–108). Apart from gender, the clinical parameters play a role in the prediction of hantavirus disease severity. For example, patients with low blood platelet count (<60G/l) usually suffer from severe acute renal failure characterized by high creatinine levels in the serum (>620 μM/l) (5, 109). The discovery of 23 hantavirus species and their broad host ranges have potentially elevated the future risks of broad-spectrum epidemics among populations.

### Diagnosis of Hantavirus Infection

The serologic tests detecting IgM and/or IgG antibodies to hantavirus antigen are most commonly used for the diagnosis of HFRS and HCPS in suspected patients. The IgG and IgM antibodies against hantavirus N protein can be detected in all most all acute HFRS and HCPS cases upon the onset of symptoms. The recombinant N protein purified from numerous expression systems such as E.coli (110, 111), baculovirus (112), saccharomyces (113, 114), plant (115, 116)and mammalian systems (117) is used as antigen for serologic testing. All three structural proteins (N protein, glycoproteins Gn and Gc) can trigger IgM response at the onset of symptoms (15, 118, 119), however, the IgG response to glycoproteins may be delayed (120). The most common serologic method for hantavirus diagnosis is the rapid IgM capture ELISA method developed by the U.S Army Medical Research Institute of Infectious Diseases and Centers for Disease Control and Prevention (CDC) (121). The test can be completed in 4–6 h (27). The rapid IgM immunochromatography strip test is commercially available for diagnosis of hantavirus infection.

Very specific and rapid diagnostic tests have been developed based on the identification of viral genome in the infected patient samples such as blood, serum, or tissue samples. This sensitive assay can detect the hantavirus infection from day one after the onset of symptoms. However, there are reports that identification of viral genome in infected patient samples can be detected before the first day of the onset of symptoms (122, 123). At this stage, the viral genome can be detected before the appearance of IgM against hantavirus antigens (124). The assay involves the reverse transcription of the viral genome from the patient samples and PCR amplification of the required viral sequence using the appropriate primer set. Due to low levels of viral RNA in infected patient samples, a nested PCR may be required, using primers targeted to the genomic regions of high homology. The nested PCR approach as a diagnostic method has been developed for HTNV (125), SNV (126), and PUUV (124). Development of multiplex PCR based diagnostic approaches focused on the identification of numerous infectious pathogens from a single patient sample in a short turnaround time is required for quick diagnostic answers and initiation of counter measures to improve disease prognosis. The rapid IgM immunochromatography strip test is commercially available for the diagnosis of hantavirus infection (127). In addition, the rapid HFRS IgG/IgM combo test is also available that simultaneously detects both IgG and IgM antibodies in the serum. since patients develop higher titers of IgM antibody at the time of clinical presentation, the rapid IgM test is more reliable for the detection of acute infection.

#### Differential Diagnosis

It is important to include leptospirosis and hantavirus infection in the differential diagnosis of acute renal failure (128). Both leptospirosis and HFRS present with classical flu-like symptoms and may be complicated by thrombotic microangiopathy with hemorrhagic phenomena and hepatic and pulmonary involvement (128). However, Jaundice should alert the physician to icteric leptospirosis (128). In high-risk areas, HFRS should be included in the differential diagnosis of acute renal failure of uncertain cause associated with febrile illness, hemorrhagic phenomenon, renal or hepatic dysfunction (129). In addition, the differential diagnosis of hantavirus induced HFRS should include spotted fevers, murine typhus, malaria, hepatitis (non-A, non-B), Colorado tick fever, septicemia, heat shock, leptospirosis, hemolytic uremic syndrome, acute abdominal disease and acute kidney injury (129).

### Vaccines Against Hantaviruses

In the United States and Europe, there is no FDA approved vaccine or antiviral therapeutic available for any of the hemorrhagic fever viruses including hantaviruses causing HFRS or NE or HCPS. Thus, except for supportive care, there is no treatment for hantavirus infection at present. However, in Korea, an inactivated hantaan virus vaccine (Hantavax^TM^) was developed that was put into commercial production in 1990 (130). Although a three dose schedule of this inactivated vaccine showed 90.14% seroconversion in phase III clinical trial, there was no statistically significant protective effect on HFRS patients (131). In China, a bivalent inactivated vaccine against the Hantaan virus and Seoul virus was produced in 1994 that was approved by the Pharmacopeia of China in 2005 (132). Under the expanded immunization program against HFRS by the government of China, approximately 2 million doses of HFRS inactivated bivalent vaccine are used annually (132, 133). HFRS cases have dropped in China after the introduction of an inactivated bivalent vaccine, suggesting the induction of effective humoral immunity that can be maintained up to 33 months after vaccination (132, 133).

The previous research focus was to develop a DNA vaccine against HFRS and HCPS (134). The focus was to express the hantavirus M protein from a plasmid harboring the M gene. Plasmid DNA based vaccines have advantages as they can't replicate or restore virulence and can't spread to the environment (93, 134). Numerous plasmids expressing the M protein from several hantavirus species were developed by the Hopper's group and tested for the development of neutralizing antibody response in Syrian hamsters [Reviewed (93)]. During vaccination, the plasmid DNAs were introduced into the host by a gene gun approach (93, 134). The M gene was cloned in the expression vector WRG 7077 and the resulting plasmids were introduced into hamster and non-human primate models, followed by the evaluation of antibody response (135). Interestingly the expression of Hantaan virus M gene was protective against Hantaan, Seoul and Dobrava virus infections in the hamster model (136). The Rhesus monkeys inoculated with plasmid (pWRG/ANDV-M) expressing the Andes virus M gene, using a gene gun approach, developed higher levels of neutralizing antibodies, and the resulting monkey serum protected 100% of infected hamsters from the fatal hantavirus disease (137). Hoppers's group has used different combinations of plasmids to determine whether simultaneous expression of M gene from different hantavirus species generates a broad immune response protective against multiple hantavirus species. Interestingly, a mixture of plasmids targeting a total of four HCPS and HFRS viruses triggered neutralizing antibodies against all four of them (138). Thus, the plasmid DNA vaccine technology against hantaviruses has created hope for the development of FDA approved vaccine against hantaviruses. The Andes virus DNA vaccine entered clinical trains in 2019. The DNA vaccine trials against HTNV are under way (139).

#### mRNA Vaccine for HFRS

The groundbreaking new approach to produce mRNA vaccine against SARS-CoV-2 by biopharmaceutical industries (Pfizer and Moderna) in 2020 has given a new direction to the general field of vaccinology and have created new hope for the rapid production of vaccines using this technologically advanced approach. The mRNA vaccines have multiple advantages compared to traditional subunit vaccines, killed and live attenuated viruses, as well as DNA-based vaccines. These advantages include safety, efficacy, and rapid production (140). The mRNA is a non-infectious, non-integrating platform, there is no potential risk of infection or insertional mutagenesis (141). The mRNA is degraded by the host RNA degradation machinery and thus the half-life of synthetic mRNA can be regulated by the chemical modification of constituent nucleotides and the modification of the delivery system used (140–142). The high efficacy of the mRNA vaccine is achieved by various modifications of the synthetic mRNA, increasing its stability and translatability. Due to the high yield of *in vitro* transcription reactions, the mRNA vaccines have the potential for rapid and inexpensive scalable manufacturing. The Conventional mRNA-based vaccines, such as Pfizer and Moderna mRNA vaccine for SARS-CoV-2, encode the antigen of interest and contain 5′ and 3′ untranslated regions (UTRs), a 5′ cap and a 3′ poly A tail (143–145). The mRNA is synthesized *in vitro*, followed by purification by chromatographic methods such as reverse-phase fast protein liquid chromatography (FPLC) or high-performance liquid chromatography (HPLC) (140). The purified mRNA can be administered with or without a career using a proper delivery approach to enhance the efficacy (140). Since the hantavirus M gene encoding the surface glycoproteins has been the focus of the vaccine development for hantaviruses (93). It is possible to transcribe the M gene encoding the glycoprotein by an *in vitro* transcription system. The mRNA can be engineered to harbor 5′ and 3′ UTRs, known to increase the mRNA translation, along with a 5′ cap and a 3′ poly A tail of appropriate length. The mRNA can be codon optimized, chemically modified by incorporating modified nucleotides during synthesis, followed by chromatographic purification to remove the double strand RNA contaminants. Strikingly, purification by fast protein liquid chromatography (FPLC) has been shown to increase protein production from *in vitro* transcribed mRNA by up to 1,000.fold in primary human DCs (146). The purified mRNA can be tested for immunological response in animal models, followed by optimization until the appropriate efficacy is achieved. Vaccination seems to be a viable approach to prevent this zoonotic infection in at least endemic areas or individuals with a higher risk for hantavirus exposure. The current vaccination efforts focused on glycoproteins (139), which elicit a protective neutralization response (137, 147–150), have created hope for the development of the hantavirus vaccine.

#### Vaccination Strategy

Hantavirus vaccine development must also be viewed from a geographical perspective. A universal hantavirus vaccine will have to consist of several antigenic components to cover for all pathogenic hantaviruses. After testing in animal models, human clinical trials should be carried out in areas with a higher prevalence of hantavirus infection. Once a safe vaccine is developed, its distribution among the population might be a challenge, people may remain less interested in vaccination due to the relatively low incidence of hantavirus infection worldwide. However, the vaccination strategy should consider priorities based on disease susceptibility, age, immunity, and chances for higher virus exposure such as populations living in rural areas or health care professionals working in hospital settings.

### Therapeutics for Hantavirus Infection

Hantaviruses primarily infect the endothelial cells of various body organs especially the kidney and lungs. The basic pathological feature of HFRS is the increased vascular permeability whose pathogenesis involves high viral load and excessive immune response of the host. Excessive capillary leakage can lead to hypotensive shock during HFRS. There are no FDA approved post-exposure therapeutic interventions for HFRS. However, several anti-viral drug development strategies have focused to interrupt the virus attachment to the host cell or disrupt the post entry steps of the viral replication cycle (Figure 4). Although some of these countermeasures (Table 3) have shown protective effects *in vitro*, none of these countermeasures are approved by FDA in the United States for clinical use. In addition, the countermeasure targeting the host system is designed to improve vasculature functions and rebuild immune homeostasis. Ribavarin, a nucleoside analog, has shown antiviral activity in both *in vitro* and *in vivo* studies against the members of Bunyavirales (27). Studies on hantavirus infected patients in China, suffering from acute kidney injury, has revealed that ribavarin therapy starting before the end of the first week of illness reduces the chances of death by seven fold (168, 169). However, ribavarin therapy on HCPS patients did not show any promising results. It was observed that 71% of HCPS patients receiving intravenous ribavarin became anemic and 19% underwent transfusion, suggesting that the efficacy of ribavarin for the treatment of HCP is questionable (170–172). The efficacy of ribavarin as a treatment for hantavirus induced AKI may depend upon the severity of the disease at the time of first administration (27). This is supported by recent observations that early intravenous treatment of ribavarin in hantavirus infected patients reduced the occurrence of oliguria and severity of renal insufficiency (173). Recently a high throughput screen identified lead compounds targeting the hantavirus N protein (162). Identification of these compounds has created new possibilities for the development of anti-hantaviral therapeutics. The passive transfer of monoclonal antibodies or polyclonal sera to HTNV or PUUV in hamsters, rats, and primates have protected these animals from hantavirus challenges (137, 174–177). A recent study suggested that a DNA vaccine /goose platform can be used to produce an antiviral biological product capable of preventing hantavirus disease when administered post-exposure (152). These observations suggest that a post-hantavirus prophylaxis treatment regime may be effective (178). New treatment strategies focused on the inhibition of virus replication and rapid prevention of vascular leakage in infected patients are urgently needed to prevent the high fatality rates in HCPS and HFRS patients. Elucidation of molecular mechanism and identification of viral and host factors involved in hantavirus induced endothelial cell dysfunction and increased vascular permeability will reveal novel targets for the design of therapeutic molecules to prevent hantavirus induced vascular leakage. Similar approaches to identify host and viral factors playing key roles in the virus replication cycle will provide avenues for the development of antiviral therapeutic agents (Figure 3). Some of the well characterized therapeutic targets, such as, the interaction between hantavirus glycoprotein and the host cell receptor, the interaction between N protein and viral genomic RNA, the interaction between N protein and RdRp, the cap snatching endonuclease and polymerase activities of the RdRp (Figure 3) can be used for the development of antiviral therapeutics. Nonetheless, the combined therapies targeting both virus replication and vascular leakage will likely improve the prognosis of this zoonotic illness. Finally, the control of animal reservoirs and the advice to populations living in endemic areas to limit the risk of exposure will significantly contribute to the preventive measures of this viral illness.

**Table 3:** Some of the therapeutic countermeasures against hantavirus induced HFRS, tested in cell culture or animal models.

**Therapeutic type**	**Target**	**Mechanism of action**	**Virus**	**Model used**
Human MAbs (Fab fragments)	Viral Gc	Blocks viral entry	PUUV	Cell culture (151)
Goose PAbs (Igγ/∧Fc)	Viral GP	Blocks viral entry	ANDV	Syrian Hamsters (152)
Rat PAbs (serum)	Viral GP	Blocks viral entry	SEOV	New born rats (153)
Mice MABs	Gc/NP	Blocks viral entry	HTNV	Mice/cell culture (154)
Lactoferin	Viral GP/host	Blocks viral entry	SEOV	Cell culture/mice (155)
Peptides (stem III)	Viral Gc	Blocks viral entry	ANDV/PUUV	Cell culture (156)
Peptidomimetic compounds	Host Receptor	Blocks viral entry	ANDV/HTNV	Cell culture (157)
Nucleoside analogs (Ribavirin)	RdRp	Virus replication	PUUV/HTNV	Mice (158–160)
Nucleoside analogs (ETAR)	RdRp	Virus replication	HTNV	Cell culture (161)
Small molecule inhibitors (K31)	NP	Virus replication	ANDV	Cell culture (162)
Small molecule inhibitors (Arbidol)	Unknown	Virus replication	HTNV	Cell culture (163)
siRNA	Viral genome	Virus replication	HTNV	Cell culture (164)
Ang-1 and S1P	Host	Improves vascular functions	HTNV/ANDV	Cell culture (165)
Corticoids or methylprednisolone	Host	Hormone (immunotherapy)	HTNV	Clinical trial (166, 167)

### Kidney Injury by Non-hemorrhagic Fever Viruses Might Provide Insight Into the Hantavirus Induced HFRS That Leads to AKI

Non-hemorrhagic fever viruses such as HIV are known to induce kidney disease. Although retroviral therapies have improved the outcome of HIV infection, the patients living with HIV remain at higher risk for chronic kidney disease due to frequent exposure to nephrotoxins. The kidney biopsies of patients with HIV associated nephropathy (HIVAN) reveal focal glomerulosclerosis and tubular cyst formation with tubulointerstitial inflammation, although such phenotypes may be more severe in patients having widespread use of combination antiretroviral therapy (cART) (179, 180). Such distinct pathologies have not been reported in hantavirus induced AKI. Numerous studies carried out in *in vitro* systems (181–183) and transgenic animal models (179, 184) have demonstrated that HIV can infect glomerular and tubular epithelial cells, and renal expression of HIV genes plays a key role in HIVAN pathogenesis. The expression of HIV transgene lacking *gag* and *po*l genes have been reported to develop kidney disease in rats and mice, showing clinical and pathological resemblance with HIVAN. Since *gag* and *pol* play crucial roles in virus replication, these studies suggest that virus replication is not necessary for HIVAN pathogenesis (184, 185). Further studies in transgenic mice showed that expression of HIV genes *vpr* and *nef* in podocytes induce glomerular disease resembling HIVAN (179, 186). The mechanism by which *vpr* induces podocyte injury remains unclear. However, *nef* is known to induce podocyte differential and proliferation by activating MAPK1,2 and Stat3 signaling pathways (187). The knockout out of Stat3 in podocytes has been reported to ameliorate the HIVAN phenotype in HIV transgenic mice (188). Similarly, *in vitro* studies have revealed that HIV *tat* gene expression induces podocye injury (189). Recent studies have demonstrated the role of Notch signaling and renin angiotensin system in podocyte injury and progression of kidney disease in HIVAN (190–192). This is supported by the amelioration of the HIVAN phenotype in animal models using chemical inhibitors targeted to these pathways (193, 194). In comparison to HIV, it is still unclear whether hantavirus replication or the expression of individual hantaviral genes is sufficient to induce AKI. Inflammatory responses have also been reported to play a role in HIVAN. The noticeable up-regulation of Kappa-B regulated proinflammatory mediators in HIV infected tubular epithelial cells and podocyte in HIVAN models suggested Kappa-B as a target molecule for therapeutic intervention of HIVAN (195, 196). Interestingly, the use of Kappa-B inhibitors ameliorated the HIVAN phenotype in HIV transgenic mice (197, 198). The molecular mechanism by which these viral factors induce kidney injury in HIV patients will help to identify targets for therapeutic intervention of HIVAN. HIV positive people harboring two copies of the APOL1-risk allele are at more risk of developing HIVAN without the use of retroviral inhibitors as compared to HIV positive people having zero or one risk alleles (199). Thus, genetic susceptibility plays a role in kidney injury induced by both HIV and hantavirus infections. Antiretroviral therapies especially nucleoside and nucleotide analogs targeting reverse transcriptase such as tenofovir, adefovir, cidofovir are all capable of inducing renal tubular injury (200, 201). AKI due to acute tebular obstruction and chronic tubulointerstital nephrits by indinavir has limited its use as an antiretroviral drug (202). Thus, while developing antivirals for hantaviruses, it is necessary to pay attention to the possible kidney injury resulting from the use of antivirals, which might worsen kidney disease.

## Conclusion

Multidisciplinary research studies have provided insights about host mechanisms such as inflammatory responses, endothelial dysfunction, oxidative and ER stress in kidney injury. Virus infection alters the host gene expression and disturbs numerous molecular pathways that may collectively contribute to kidney injury in infected hosts. Although the overwhelming immune response plays a major role in hantavirus disease (Figure 4), it is still difficult to draw a detailed mechanistic picture for the pathogenesis of hantavirus induced AKI. Identification of viral and host factors such as gender, HLA haplotype, viral load, and inflammatory response have helped physicians to predict the clinical outcome of the disease. Analysis of vascular leakage has revealed the breakdown of the endothelial cell barrier by the impairment of cell-to-cell contact. The loss of cellular contact in the endothelium may be due to disturbances in signaling pathways involving vascular endothelial growth factor, E-cadherin, and kallikrein-kinin system (Figure 4). Identification and characterization of host factors mediating the vascular leakage during hantavirus infection will provide crucial insights for the development of therapeutic strategies to prevent vascular leakage and improve the prognosis of hantavirus disease. Combinational therapeutic approaches aimed at inhibiting both virus replication and vascular leakage would likely have a better outcome. AKI induced by old word hantaviruses has a good prognosis at present, both in the long and short term. However, hantaviruses are continuously evolving due to mutations in the genome by RdRp, which is deficient in proof-reading activity. The emergence of future virulent strains with the potential to cause severe AKI with a bad prognosis cannot be ruled out. This is supported by the emergence of hantavirus cases in Asia and Europe with clinical manifestations resembling new world hantaviruses and vice versa (203, 204). Thus, the development of potential vaccines and antiviral therapeutics is necessary to keep this zoonotic illness under control. Due to the lack of vaccine and antiviral therapies, preventive measurements such as closer attention of endemic areas, control of mice inside and outside of homes, and prevention of contact with contaminated aerosols is the only way to reduce hantavirus disease mortalities.

## Author Contributions

The author confirms being the sole contributor of this work and has approved it for publication.

## Funding

This work was funded for publication of this article will be provided from the startup funds provided to the author by the Western University of Health Sciences.

## Conflict of Interest

The author declares that the research was conducted in the absence of any commercial or financial relationships that could be construed as a potential conflict of interest.

## Publisher's Note

All claims expressed in this article are solely those of the authors and do not necessarily represent those of their affiliated organizations, or those of the publisher, the editors and the reviewers. Any product that may be evaluated in this article, or claim that may be made by its manufacturer, is not guaranteed or endorsed by the publisher.
